# Patient-reported experiences of cancer care related to the COVID-19 pandemic in Switzerland

**DOI:** 10.1007/s00520-023-07871-8

**Published:** 2023-06-22

**Authors:** Sara Colomer-Lahiguera, Claudia Canella, Stellio Giacomini, Kim Lê Van, Carla Pedrazzani, Matthias Naegele, Laure Thouvenin, Alix O’Meara Stern, Rosaria Condorelli, Tourane Corbière, Claudia M. Witt, Manuela Eicher, Karin Ribi

**Affiliations:** 1 Institute of Higher Education and Research & Department of Oncology Faculty of Biology and Medicine, University of Lausanne and Lausanne University Hospital, Office 01/169 - PROLINE - Rte de la Corniche, 10–1010 Lausanne, Switzerland; 2 Institute for Complementary and Integrative Medicine, University Hospital Zurich and University of Zurich, Zürich, Switzerland; 3 Charité – Universitätsmedizin Berlin, Corporate Member of Freie Universität Berlin, Humboldt-Universität zu Berlin, and Berlin Institute of Health, Institute of Social Medicine, Epidemiology and Health Economics, Berlin, Germany; 4 Department of Economics, Health and Social Sciences, University of Applied Sciences and Arts of Southern Switzerland, Manno, Switzerland; 5 Cantonal Hospital St. Gallen, Department of Development and Quality Management in Nursing, Network Oncology, St. Gallen, Switzerland; 6 Comprehensive Cancer Center Zurich, University Hospital Zurich, Zürich, Switzerland; 7 Department of Oncology, University Hospitals of Geneva (HUG), Geneva, Switzerland; 8 Department of Medical Oncology, Réseau Hospitalier Neuchatelois, Neuchâtel, Switzerland; 9 Department of Medical Oncology, EOC - Istituto Oncologico della Svizzera Italiana, Bellinzona, Switzerland; 10 Department Health, Kalaidos University of Applied Sciences, Zürich, Switzerland

**Keywords:** COVID-19, Distress, Resilience, Patient-reported experience, Oncology

## Abstract

**Purpose:**

This study aims to describe the experience of Swiss oncological patients during the COVID-19 pandemic.

**Methods:**

A national multi-center study including five hospitals covering the three main language regions of Switzerland was conducted between March and July 2021. Patients with melanoma, breast, lung, or colon cancer receiving active systemic anti-cancer treatment at the time of the COVID-19 pandemic were included. We conducted semi-structured telephone or onsite interviews alongside the administration of distress and resilience-validated questionnaires. Thematic analysis was performed for the qualitative data and descriptive statistics for the quantitative data.

**Results:**

Sixty-two cancer patients with a mean age of 61 (SD=14) (58% female) were interviewed. Based on the interviews, we identified that the experience of having cancer during the COVID-19 pandemic was related to five dimensions: psychological, social, support, healthcare, and vaccination. Three themes transverse the five dimensions: (a) needs, (b) positive changes, and (c) phases of the pandemic. In general, patients did not experience delays or disruptions in their cancer treatment nor felt additionally burdened by the pandemic. Lockdown and isolation were reported as mixed experiences (positive and negative), and access to vaccination reassured patients against the risk of infection and instilled hope to return to normalcy. Additionally, we found low distress levels (M=2.9; SD=2.5) and high resilience scores (M=7; SD=1.3) in these patients.

**Conclusion:**

Swiss patients with cancer did not express major needs or disruptions in their care during this period of the COVID-19 pandemic. Results identify the mixed experiences of patients and highlight the high resilience levels.

**Supplementary Information:**

The online version contains supplementary material available at 10.1007/s00520-023-07871-8.

## Introduction

At the outbreak of the COVID-19 pandemic in 2020, international and national oncology societies developed recommendations aiming to re-organize the functioning of cancer centers such as limiting hospital visits, reducing admissions, and decreasing treatment administration without compromising patient outcomes [1]. However, adjustments on care delivery translated into delays and cancellations of follow-up appointments, surgeries, or screening programs and the replacement of in-person interactions with telehealth consultations [2–4]. These changes magnified uncertainty [5–7] and had an impact on the quality of life of patients with cancer [8–11].

Major topics identified by people affected by cancer were concerns related to the impact of COVID-19 on cancer care, adaptation challenges to the new context, or the need for information about COVID-19 and (self-)management of cancer symptoms and treatment during the pandemic [12]. In addition, fear of cancer recurrence, unmet needs, pre-existing health conditions, younger age, financial concerns, and perceived risk of contracting COVID-19 were the dominant factors contributing to psychological distress and anxiety in patients with cancer or in remission [10, 13]. Furthermore, the inability to ensure the presence of loved ones further heightened the social isolation and the feeling of vulnerability of these patients [2, 14, 15]. Anxiety prevalence during the pandemic has been reported in 20 to 50% of patients with cancer [6, 15–23]. Their distress levels varied over time with moderate to severe levels found at the beginning of the pandemic (March to June 2020) [24–27] and lower levels reported after this period [18, 28–30]. In contrast, resilience levels remained high across the different phases of the pandemic [22, 28, 30].

Cancer centers in Switzerland adopted initial emergency protocols based on international recommendations to minimize the risk of infection for both patients and healthcare professionals. The impact of these measures on cancer care in central Europe has been documented [31–33].

Given the impact of the COVID-19 pandemic on psychological burden and cancer care, it is important to understand how patients with cancer navigated the pandemic and the specific challenges they encountered, to ensure adequate support. This national study aims to describe, for the first time, the experience of Swiss patients with cancer during the COVID-19 pandemic and to explore their distress and resilience levels.

## Methods

### Design

We developed a national multi-center descriptive study employing qualitative (semi-structured interviews) complemented by quantitative methods (questionnaire) to investigate the experiences of cancer patients under active treatment during the COVID-19 pandemic. The study has been replicated with adaptations in several European countries [28, 30] and in the USA. The consolidated criteria for reporting qualitative studies (COREQ) was used as a reporting guideline [34].

### Recruitment and sample

Patients were recruited in five hospitals (three universities and two tertiary centers) covering the three main language regions of Switzerland including French (CH-FR), German (CH-DE), and Italian (CH-IT). Adult patients diagnosed with a melanoma, breast, lung, or colon cancer were approached via the clinical or research team during their visits to the hospital and invited to participate. We included patients receiving active systemic anti-cancer treatment (oral or intravenous, including chemotherapy, targeted therapy, and immunotherapy) with adjuvant or palliative intent at the time of the COVID-19 pandemic; patients receiving adjuvant or palliative cancer care; and able to speak French, German, or Italian. Exclusion criteria included hospitalized patients, patients in an end-of-life situation; patients currently receiving radiotherapy or surgical treatment (even if combined with systemic therapy), patients who have or had laboratory-confirmed infection with SARS-CoV-2 virus or clinically/radiologically diagnosed COVID-19 infection, and patients not able to follow study procedures.

### Data collection

Semi-structured interviews were conducted either by telephone or on-site, depending on the availability of the patient, in French, German, or Italian. Participants were asked about their experiences during the pandemic and how they were affected in their illness trajectory (Online material Table 1). Interviews had an average duration of 35 min.

At the end of the interview, participants were invited to respond to a brief questionnaire about demographic characteristics (age, gender, responsibilities as caregiver), their distress, and resilience levels. Distress was measured using the National Comprehensive Cancer Network Distress Thermometer (NCCN-DT), a one-item visual analog scale (0, “no distress,” to 10, “extreme distress”) used to screen for self-reported psychosocial distress in different cancer settings [35]. The 2-item Connor-Davidson-Resilience Scale (CD-RISC-2) [36] was used to evaluate participants’ resilience via two items selected by the originators of the scale as etymologically capturing the essence of resilience: “able to adapt to change” and “tend to bounce back after illness or hardship” and measured with a 5-point Likert scale (0, “not at all true,” to 4, “true nearly all of the time”). An overall CD-RISC-2 score is calculated from the sum of both items (range of 0–8) with higher scores reflecting higher levels of resilience. Patients’ clinical characteristics, including cancer diagnosis, current cancer treatment, and comorbidities, were extracted from the electronic health record of the patient.

Interviews took place in all linguistic regions from March to July 2021 (CH-FR: 24.03.2021–18.07.2021; CH-DE: 30.03.2021–27.07.2021; CH-IT: 25.03.2021–06.07.2021). This interval corresponded to the period after the second wave of the COVID-19 pandemic that reached its peak in Switzerland between November and December 2020. Compared to the first wave (March 2020), the number of confirmed cases and deaths was higher. However, hospital capacity was stabilized at the time of data collection [37]. From the end of February 2021, control measures were progressively eased. Testing strategy was extended, and free self-tests were available. Private outdoor gatherings as well as publicly accessible recreational and entertainment establishments were again permitted with a restricted number of attendants, and many previous requirements became recommendations (e.g., homeworking, mask-wearing) [37].

### Data analysis

A thematic analysis was conducted to identify, analyze, and report patterns (themes and sub-themes) [38] using MAXQDA software. The different patterns were identified in an analytic process based on the interview data and visualized at different thematically aggregated levels, starting with the most detailed level of codes, which were then grouped thematically under subthemes. The subthemes were then further aggregated at a higher thematic level to form themes which in their nature describe *dimensions* of the patients’ experiences of having cancer during the COVID-19 pandemic. We use the term “dimensions” to describe the nature of the themes. In detail, we applied a deductive semantic initial code approach to the raw qualitative data based on results from our previous analysis of online forums where we identified three major themes: (1) concerns related to the impact of COVID-19, (2) adaptation challenges on the individual and societal level, and (3) the need for advice [12]. This was complemented with an inductive process creating additional codes based on the interview data. First, three random transcripts per language region were analyzed independently to generate a preliminary codebook. Then, an analysis team of multi-lingual researchers (SG, KLV, KR, SCL for the French; CC and MN for the German; and CP and KR for the Italian) met regularly to discuss and refine the codebook on a national level and to identify regional commonalities and differences. The codes were then collapsed into subthemes and themes in an intersubjective validation and synchronization process among all study sites. Lastly, themes and subthemes were discussed with two clinicians (oncologists) and a patient representative to ensure the trustworthiness of the results.

Patients’ distress and resilience scores as well as socio-demographic and clinical data were analyzed descriptively using a standard software package (Stata, version. 17.0; StataCorp).

## Results

### Characteristics of participants

Sixty-two patients with breast (33.9%), lung (25.8%), colon (25.8%) cancer, or melanoma (14.5%) were interviewed. The mean age was 60.9 (SD=14) with women representing 58% of the sample. Gender distribution varied across the three regions. Most of the participants lived with their partners and did not have children and/or relatives in need of care living in the same household. Most of the patients (85.5%) were on ongoing systemic treatment at the time of the interview, and 40% had at least one comorbidity (Table 1).

**Table 1:** Socio-demographic and clinical characteristics of the participants

Variables		All regions *N*=62 *n* (%)	CH-FR *N*=35 *n* (%)	CH-DE *N*=18 *n* (%)	CH-IT *N*=9 *n* (%)
Age – mean (SD)		60.9 (14)	64.3 (12.8)	53.3 (13.5)	62.4 (15.1)
Gender	Male	26 (41.9)	20 (57.1)	3 (16.7)	3 (33.3)
	Female	36 (58.1)	15 (42.9)	15 (83.3)	6 (66.7)
Living situation	Single/living alone	12 (19.4)	7 (20)	4 (22.2)	1 (11.1)
	Living with partner	40 (64.5)	21 (60)	12 (66.7)	7 (77.8)
	Living separated from partner/husband/wife	7 (11.3)	4 (11.4)	2 (11.1)	1 (11.1)
	Widowed/partner deceased	3 (4.8)	3 (8.6)	-	-
Children and/or relatives in need of care living in the same household	Yes	9 (14.5)	5 (14.3)	3 (16.7)	1 (11.1)
	No	52 (83.9)	30 (85.7)	14 (77.8)	8 (88.9)
	Missing	1 (1.6)	0	1 (5.6)	0
Education	No degree	2 (3.2)	2 (5.7)	-	-
	Compulsory education	6 (9.7)	5 (14.3)	-	1 (11.1)
	Vocational training	20 (32.3)	11 (31.4)	4 (22.2)	5 (55.6)
	Higher technical education/university of applied sciences	20 (32.3)	8 (22.9)	10 (55.6)	2 (22.2)
	University	14 (22.5)	9 (25.7)	4 (22.2)	1 (11.1)
Current main professional activity	Employed	17 (27.4)	10 (28.6)	4 (22.2)	3 (33.3)
	Self-employed	6 (9.7)	2 (5.7)	4 (22.2)	-
	Retired	25 (40.3)	15 (42.9)	4 (22.2)	6 (66.7)
	Homemaker	2 (3.2)	1 (2.9)	1 (5.6)	-
	Disability due to illness or accident	11 (17.7)	6 (17.1)	5 (27.8)	-
	Other	1 (1.6)	1 (2.9)	-	-
Cancer type	Breast	21 (33.9)	8 (22.9)	8 (44.4)	5 (55.6)
	Lung	16 (25.8)	13 (37.1)	2 (11.1)	1 (11.1)
	Colon	16 (25.8)	8 (22.9)	5 (27.8)	3 (33.3)
	Melanoma	9 (14.5)	6 (17.1)	3 (16.7)	-
Currently receiving systemic treatment	Yes	53 (85.5)	29 (82.9)	15 (83.3)	9 (100)
	No	9 (14.5)	6 (17.1)	3 (16.7)	-
Comorbidities	Diabetes mellitus	4 (6.5)	2 (5.7)	1 (5.6)	1 (11.1)
	Heart failure	5 (8)	4 (11.4)	-	1 (11.1)
	Mental health illness	1 (1.6)	-	1 (5.6)	-
	Other comorbidities	16 (25.8)	9 (25.7)	6 (33.3)	1 (11.1)

### Patients’ experience

**Fig. 1:**
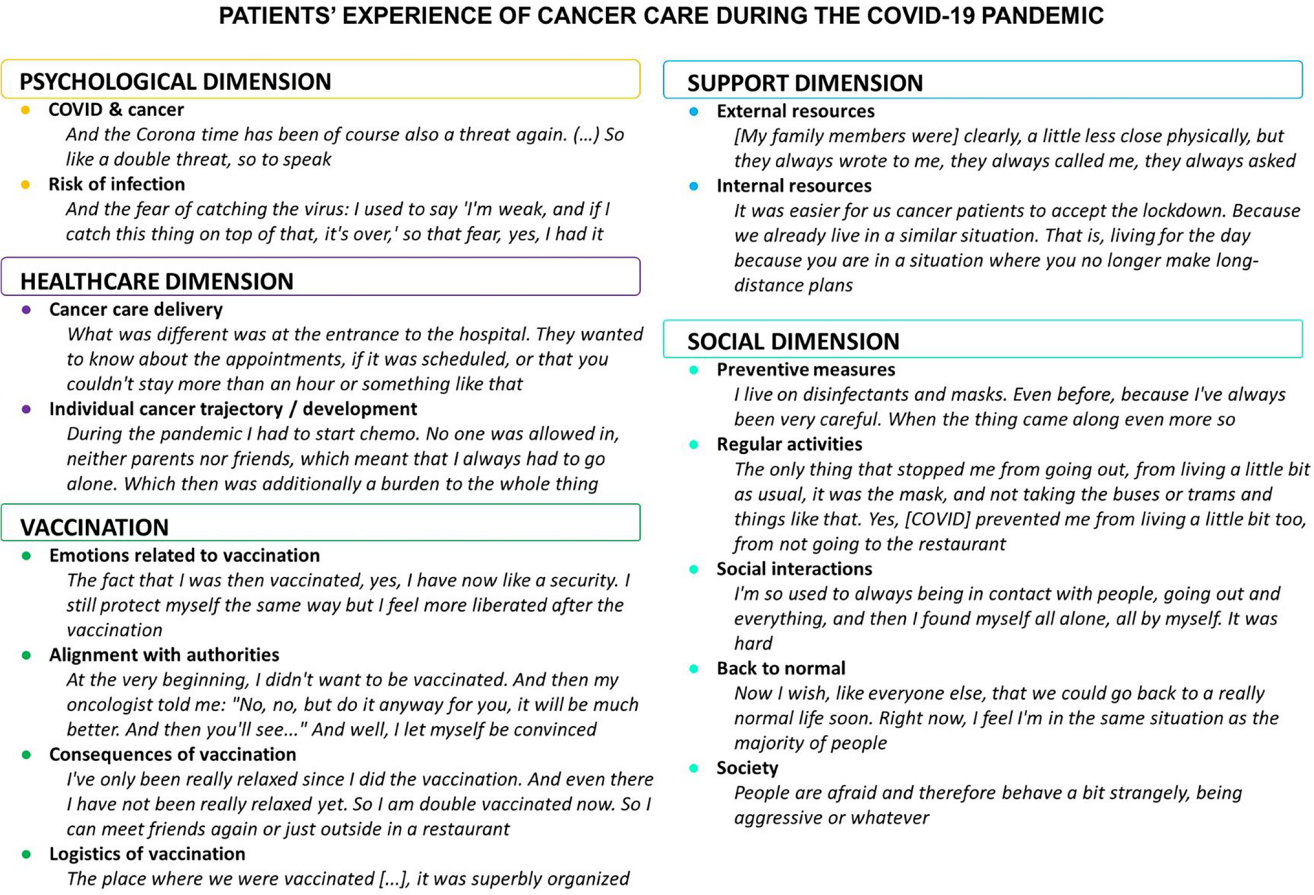
Thematic map representing the five themes reflecting the different dimensions and subthemes identified with selected quotes. Codes for each subtheme and illustrative quotes for each of the regions can be found in the Online material Table 2. The interviews were conducted in the native languages of the three regions: French, Swiss German, and Italian. The quotes were then translated to English by the authors

Overall, the majority of patients with cancer did not express significant consequences regarding the impact of the COVID-19 pandemic on their disease management or care. We generated five dimensions of patients’ experience of having cancer during the COVID-19 pandemic: (i) psychological, (ii) social, (iii) support, (iv) healthcare, and (v) vaccination (Fig. 1). A detailed table with the themes (dimensions), subthemes, their definitions, codes, and representative quotes for each of the regions is provided as an Online material Table 2.

#### Psychological dimension

The psychological dimension gathers two thematic subthemes: *psychological or emotional state related to COVID and cancer* and the perception of the *risk of infection*. Although the majority reported not having experienced a double burden of having cancer during the COVID-19 pandemic, a number of patients acknowledged having concerns about the impact of the pandemic on their cancer disease, on their loved ones, the feeling of isolation, and the general uncertainty regarding the situation. Some patients expressed the feeling of being more vulnerable, hence at risk, to COVID-19 infection because of their cancer and treatment*.* For some patients, the hospital even became a safer place due to all the measures put in place.

Patients strongly related the psychological dimension to the social dimension as they perceived having to execute a constant benefit-risk tradeoff between their own mental health and the risk of infection, especially when it came to their need for cultivating relationships, social interactions, and regular activities around them. However, patients also highlighted that all the preventive measures around the pandemic and ultimately the vaccination reduced their worries about risking a COVID-19 infection.

#### Social dimension

The social dimension gathers five thematic subthemes of major changes that patients experienced as a consequence of the pandemic: *preventive measures*, *regular activities*, *social interactions*, *back to normal*, and the impact on *society’s behavior*.

*Preventive measures* (including hygienic measures, social distancing, lockdown, or avoiding physical contact) and *regular activities* were two main aspects of the social dimension. In general, patients felt reassured when following the recommendations. While control measures such as *social distancing* and *lockdown* were difficult to deal with for some patients, others felt relieved and safer staying at home and in isolation. Nevertheless, these changes were generally accepted as part of a new reality and transitioned into a new way of life.

Patients frequently experienced a lack of *social contact* and change in *social interaction* often linked to the closing of social places replaced by digital communication, and many expressed the wish to *get back to normalcy*, to the life they had before the pandemic. Finally, patients reported seeing a change in *society.* This change was perceived in two ways: while some patients mentioned experiencing a newfound solidarity, others observed a collective behavior towards a more pronounced individualism.

#### Support dimension

Support was a recurrent theme in patients’ discourse, organized in terms of external and internal resources. *External resources* includes any statement that relates to a support received by an informal caregiver, community, or non-institutional support. Most patients reported feeling well-supported by relatives and friends during the pandemic. *Internal resources* relates to any state of mind, resilience, coping strategies, or activities helping the patient during the pandemic. Overall, patients showed great resilience. Some patients connected this resilience to their experience of living with cancer.

#### Healthcare dimension

Within the healthcare dimension, statements related to *cancer care delivery* and the *individual cancer trajectory*. C*ancer care delivery* includes the reorganization of cancer centers, cancer center measures taken, and the feeling of support from the clinical team and shows different characteristics and qualities identified by participants as helpful, challenging, positive, or negative. Most of the patients did not experience many delayed appointments, but mostly a reorganization of their clinical visits, and felt supported and accompanied by the clinical team. In general, patients reported no impact of the pandemic on the *individual cancer trajectory*, namely consequences of changes in patients’ individual illness trajectory. While many patients reported suffering from loneliness due to the no-visit rule, it was also mentioned that they enjoyed the quietness and slow pace. For instance, for some patients, it was kind of a relief not to be accompanied while receiving their chemotherapy treatment so that they did not have to explain to their families or friends that they prefer to be alone during their visits at the clinic.

#### Vaccination

Experience of vaccination is organized around four thematic subthemes: *emotions related to vaccination*, *alignment with authorities*, the *consequences of vaccination*, and the *logistics of vaccination*. Patients talked about *emotions and perceptions of vaccination*, reporting expectations, hopes, and fears about vaccination referring to oneself or to other people. Most of the patients expressed a positive perception of the vaccination, feeling reassured or protected after being vaccinated, and leading to a sort of “liberation.”

We identified the *alignment with authorities* as a main component of patients’ experience. Despite initial doubts related to the quick development and lack of evidence of vaccines’ effectiveness, many patients indicated their overall trust in health policies, following advice mainly from their oncologist, and accepted the “unavoidable” vaccination. Patients mentioned the *consequences of vaccination*, including the impact on individual, public health, and non-health domains; symptoms; and the self-protection and the protection for the others. Likewise, *logistics of vaccination*, namely organization, procedures, or appointments, is a theme succinctly but frequently mentioned by patients.

#### Transversal themes

Three themes were attributed across dimensions. These themes derived from specific questions included in our interview guide and related to (i) the needs that patients might have linked to the pandemic and their disease, (ii) changes due to the pandemic that the patient would experience as positive, and (iii) changes during the different phases of the pandemic (Online material Table 3) and were not considered a dimension as they occurred in more than one dimension (e.g., psychological and social dimension)

In general, patients stated that they did not have specific needs linked to the pandemic and their disease aside from sticking to the hygienic measures to be better protected. They expressed more likely the need for social interactions and regular activities independently from being a person affected by cancer.

As immunosuppressed and fatigued, patients felt supported by the hygienic measures, mandatory masks, social distancing, and home office during the pandemic. In addition, as the cultural and social life was restricted for everybody during the pandemic, patients with cancer felt that they missed out less and needed to explain less towards others about their restrictions.

The most obvious change during the different phases of the pandemic was the availability of vaccination. Most of the patients felt less at risk for infection after being vaccinated and started to feel more at ease with social interactions.

### Distress and resilience

Distress levels were on average low (mean=2.9, SD=2.5), with some differences between regions. While the French-speaking part showed the lowest scores (M=2, SD=2.1), the German- and especially the Italian-speaking part reported an average of 4.1 (SD=2.5) and 4.2 (SD=2.9), respectively (Table 2). Resilience scores were generally high (M=7; SD=1.3) without much variation between regions (Table 2).

**Table 2:** Distress and resilience scores

	*N*	Mean	SD	Median	Min	Max
Distress (0–10)						
NCCN-DT	61*	2.9	2.5	2	0	8
CH_FR	35	2	2.1	1	0	8
CH_DE	17*	4.1	2.5	3	1	8
CH_IT	9	4.2	2.9	5	0	8
Resilience (0–8)						
CD-RISC-2	62	7	1.3	7.5	3	8
CH_FR	35	6.7	1.3	7	3	8
CH_DE	18	7.5	0.9	8	5	8
CH_IT	9	6.8	1.3	7	5	8

*1 data missing. The NCCN practice guideline recommends that a DT score of 4 or higher indicates moderate-to-severe distress and mild distress corresponds to a DT score < 4 [35]. Validation of the German version of the NCCN-DT identified a score of ≥5 at the visual analog scale as a cutoff for a clinically significant level of distress [39]

## Discussion

The aim of this study was to describe the experience of Swiss patients with cancer during the COVID-19 pandemic, between March and July 2021. Five main themes were identified from the interviews related to psychological, social, healthcare, support, and vaccination dimensions of patients’ experiences. Most of the patients did not express major needs, worries, or disruptions on their cancer care. This was supported by the results from the quantitative data showing low levels of distress and high resilience. Although in general patients followed the preventive recommendations and adapted to the new measures, the main issues identified were social distancing, the lack of personal interactions, and the limitations regarding visits. Most of the patients agreed on the vaccination as the right thing to do to feel protected and provided a light of hope to go back to normal. Despite some doubts, especially due to the rapidity of commercialization, the majority followed their oncologist’s advice to get vaccinated.

Similar to other studies, patients shared the fear of contracting the virus [13, 24, 26] or feeling vulnerable [28, 30]. As reported by Drury and colleagues [30], we also found in our sample that the establishment of control and hygienic measures, home-office, and the slowed down cultural and social life appeared more as a relief to some patients rather than an additional burden. However, a recurrent challenge on patient’s life was represented in our study by the isolation due to the social distancing and lockdown, the lack of personal interactions, and the restrictions on hospital visits. The importance of family or friends’ support was a recurrent theme as previously described in other studies [24, 28, 30]. Recent research has shown the association between higher COVID-19 policy stringency and higher mean psychological distress scores and lower life evaluations [40]. Interestingly, some patients mentioned that having cancer helped them to cope with the COVID-19 situation. Lazarus and Folkman define “coping” as the cognitive and/or behavioral efforts to deal with stressful or difficult situations [41]. In our study, patients talked about internal and external resources that helped them to overcome pandemic-related circumstances, such as lockdowns and social distancing, uncertainty, and hygienic or protective measures. For these patients, these resources seemed to be already established due to their disease and thus may explain the high levels of resilience.

Although studies reported distress associated with a cancer diagnosis or fear of cancer progression [6, 42], those themes were not identified in the Swiss population. Likewise, some studies found economic-related issues such as increased financial hardship [13] or the cost of living with cancer during the COVID-19 pandemic [28] that did not appear in our analysis. Another important topic represented in the literature, especially in early studies, has been changes on cancer services and reorganization, sometimes leading to delays or disruptions [2, 10, 12, 28]. However, in our population, this did not seem to have a prominent impact, except for the no-visit rule, especially when patients had a diagnostic announcement. Finally, communication and information needs identified by others [6, 13, 24, 42] rarely appeared in our study. One possible reason could be attributed to the time period during which the data was collected, being a relatively stable period with more information available compared to the beginning of the pandemic.

In terms of distress and resilience, our results are in line with results from the Danish [28] and Irish [30] studies having replicated the study protocol. Both studies found low distress levels (Danish: M=2.3, SD=2.6; and Irish: M=3.4, SD=2.2) and high resilience (Danish: M=7.25, SD=1.1; and Irish: M=6.5, SD=1.6). Similarly, two other studies from Brazil used the NCCN-DT to collect distress data. Mendonça and colleagues [29] measured distress in patients initiating a treatment between December 2020 and March 2021 showing comparable distress levels (M=3.81, SD=3.46), and Rodrigues-Oliveira [18] in patients with head and neck cancer between June and August 2020 reporting a mean DT score of 3.68 (SD=2.77). All these studies conducted their data collection during or after the second wave of the pandemic, a period when more information and evidence were available and vaccines started to be accessible.

### Strengths and limitations

Recruitment for this study took place in person at the different cancer centers. The study population is therefore limited to patients who accessed the hospital despite the conditions of the pandemic. These patients benefited from consultations with health professionals and access to diagnostic, tests, and treatments. By contrast, patients who might avoid visits to the hospital and thus potentially experienced delays or lack of treatment were not included in the study, but may have experienced higher distress levels.

Although the CD-RISC has been used to study resilience and mental health during the COVID-19 pandemic [43–45], a recent publication highlighted the less desirable psychometric properties of the German CD-RISC-2 compared to the CD-RISC-10 [46], recommending it only for situations when completion time is critical. Nevertheless, in our study, resilience was measured as a complement to the qualitative approach and was not the main focus of the study.

While the sample size was a strength for the main qualitative component of the study, it remained relatively small for the descriptive quantitative approach. Nonetheless, distress and resilience scores supported the results from the interviews.

Finally, results from this study may reflect a specific context in a unique and rapidly evolving situation such as the COVID-19 pandemic. Epidemiologic, social, economic, political, or healthcare system saturation at the time of data collection are factors that may influence patients’ experience and must therefore be taken into consideration when interpreting results providing a comprehensive understanding of the experiences of patients with cancer that can inform future developments of patient-reported experience measures.

## Conclusion

At the initiation of our study, the common assumption was that people with cancer would experience additional stress by the COVID-19 pandemic that would affect their resilience. In contrast, Swiss patients with cancer showed high resilience and low levels of distress and, consistently, did not experience major needs or worries related to their disease or disruptions in their cancer care during this period of the COVID-19 pandemic. Risk of infection and isolation were the two main concerns identified. With the access to vaccination, patients felt less at risk and expressed their need for going back to more social interaction and regular activities. Future research might explore how strategies of resilience and coping in patients with cancer or a chronic disease can inform interventions to better support and reinforce such strategies or resources in these population.

## Supplementary information

ESM 1 (DOCX 31 kb)

ESM 2 (PDF 413 kb)

## Data Availability

Not applicable.
