# Supplementary material for: Patient-reported experiences of cancer care related to the COVID-19 pandemic in Switzerland
Source: Support Care Cancer. 2023 Jun 22;31(7):410. doi: 10.1007/s00520-023-07871-8 (PMC10287824; doi:10.1007/s00520-023-07871-8)
Supplement: Supplementary file 1 — (DOCX 31 kb) [file 520_2023_7871_MOESM1_ESM.docx]

**Appendix: Consolidated criteria for reporting qualitative studies (COREQ): 32-item checklist**

**Patient-reported experiences of cancer care related to the COVID-19 pandemic in Switzerland**

Sara Colomer-Lahiguera^1*#^, Claudia Canella^2,3#^, Stellio Giacomini^1^, Kim Lê Van^1^, Carla Pedrazzani^4^, Matthias Naegele^5,6^, Laure Thouvenin^7^, Alix O'Meara Stern^8^, Rosaria Condorelli^9^, Tourane Corbière^1^, Claudia M. Witt^2,3^, Manuela Eicher^1^, Karin Ribi^10^

# These authors contributed equally/shared first authorship

^1^ Institute of Higher Education and Research & Department of Oncology Faculty of Biology and Medicine, University of Lausanne and Lausanne University Hospital, Lausanne, Switzerland

^2^ Institute for Complementary and Integrative Medicine, University Hospital Zurich and University of Zurich, Zurich, Switzerland

^3^ Charité – Universitätsmedizin Berlin, corporate member of Freie Universität Berlin, Humboldt-Universität zu Berlin, and Berlin Institute of Health, Institute of Social Medicine, Epidemiology and Health Economics, Berlin, Germany

^4^ Department of Economics, Health and Social Sciences, University of Applied Sciences and Arts of Southern Switzerland, Manno, Switzerland

^5^Cantonal Hospital St. Gallen, Department of Development and Quality management in Nursing, Network Oncology, St. Gallen, Switzerland

^6^Comprehensive Cancer Center Zurich, University Hospital Zurich, Zurich, Switzerland

^7^ Department of Oncology, University Hospitals of Geneva (HUG), Geneva, Switzerland

^8^ Department of Medical Oncology, Réseau Hospitalier Neuchatelois, Neuchâtel, Switzerland

^9^ Department of Medical Oncology, EOC - Istituto Oncologico della Svizzera Italiana, Bellinzona, Switzerland

^10^ Department Health, Kalaidos University of Applied Sciences, Zürich, Switzerland

| **No** | **Item** | **Guide questions/description** | **Reporting** |
| --- | --- | --- | --- |
| **Domain 1: Research team and reflexivity** |  |  |  |
| Personal Characteristics |  |  |  |
| 1. | Interviewer/facilitator | Which author/s conducted the interview or focus group? | Sara Colomer-Lahiguera, Claudia Canella, Stellio Giacomini, Kim Lê Van, Matthias Naegele, Dario Valcarenghi, Angela Tolotti, Célia Darnac, Joëlle Ka Yann Ho, Melissa Christofis, Isabelle Puricelli, Anne-Lise Paratte (formerly Schneider) and Catia Serra |
| 2. | Credentials | What were the researcher's credentials? *E.g. PhD, MD* | Sara Colomer-Lahiguera: PhD  Claudia Canella: MA  Stellio Giacomini: MSc  Kim Lê Van: PhD  Carla Pedrazzani: PhD  Matthias Naegele: MScN  Laure Thouvenin: Dr. med.  Alix O'Meara Stern: PD, Dr. med.  Rosaria Condorelli: Dr. med.  Tourane Corbière: patient partner  Claudia M. Witt: Prof. Dr. med., MBA  Manuela Eicher: Prof. Dr. rer. med.  Karin Ribi: PhD, MPH |
| 3. | Occupation | What was their occupation at the time of the study? | Sara Colomer-Lahiguera: Senior Nurse Scientist at affiliation 1  Claudia Canella: Researcher at affiliation 2; Doctoral student at affiliation 3  Stellio Giacomini: Researcher at affiliation 1  Kim Lê Van: Researcher at affiliation 1  Carla Pedrazzani: Researcher at affiliation 4  Matthias Naegele: Nurse expert APN at affiliation 6  Laure Thouvenin: Oncologist at affiliation 7  Alix O'Meara Stern: Oncologist at affiliation 8  Rosaria Condorelli: Oncologist at affiliation 9  Tourane Corbière: patient partner at affiliation 1  Claudia M. Witt: Director of affiliation 2, Head of working group integrative medicine and digital health at affiliation 2  Manuela Eicher: Professor at affiliation 1  Karin Ribi: Senior researcher at affiliation 1 |
| 4. | Gender | Was the researcher male or female? | Claudia Canella: Female  Sara Colomer-Lahiguera: Female Stellio Giacomini: Male  Kim Lê Van^:^ Female  Carla Pedrazzani: Female  Matthias Naegele: Male  Laure Thouvenin: Female  Alix O'Meara Stern: Female  Rosaria Condorelli: Female  Tourane Corbière: Female  Claudia M. Witt: Female  Manuela Eicher: Female  Karin Ribi: Female |
| 5. | Experience and training | What experience or training did the researcher have? | All the interviewers were trained on the interview guideline in November 2020 by the principal study site.  The analyses were performed by the following experienced qualitative researchers: Karin Ribi, Stellio Giacomini, Kim Lê Van, Sara Colomer-Lahiguera, Claudia Canella, Matthias Naegele, Carla Pedrazzani and Manuela Eicher. |
| Relationship with participants |  |  |  |
| 6. | Relationship established | Was a relationship established prior to study commencement? | This differed among the interviewers, as some of the health professionals already met some of the patients during their therapy while the researchers did not meet the patients prior to the interview. |
| 7. | Participant knowledge of the interviewer | What did the participants know about the researcher? e*.g. personal goals, reasons for doing the research* | What can be found on the institutional websites of the different affiliations 1-9.  We did not systematically inform the interviewees about our personal background and interests in the research, unless they asked about it. |
| 8. | Interviewer characteristics | What characteristics were reported about the interviewer/facilitator? e.g. *Bias, assumptions, reasons and interests in the research topic* | What can be found on the institutional websites of the different affiliations 1-9.  We did not systematically inform the interviewees about our personal background and interests in the research, unless they asked about it. |
| **Domain 2: study design** |  |  |  |
| Theoretical framework |  |  |  |
| 9. | Methodological orientation and Theory | What methodological orientation was stated to underpin the study? *e.g. grounded theory, discourse analysis, ethnography, phenomenology, content analysis* | The study was underpinned by a qualitative research methodology using semi-structures interviews complemented by quantitative data collected via questionnaires at the end of the interviews.  We applied thematic analyses according to Braun and Clarke 2006 (see references).  See chapter 2.1, 2.3, and 2.4 |
| Participant selection |  |  |  |
| 10. | Sampling | How were participants selected? *e.g. purposive, convenience, consecutive, snowball* | We performed a purposive sampling. |
| 11. | Method of approach | How were participants approached? e*.g. face-to-face, telephone, mail, email* | See chapter 2.2 (face-to-face, phone, and email). |
| 12. | Sample size | How many participants were in the study? | 62, see chapter 3.1 and Table 1. |
| 13. | Non-participation | How many people refused to participate or dropped out? Reasons? | 5 dropouts due to hospitalization, migration, lack of time, not reachable after informed consent. |
| Setting |  |  |  |
| 14. | Setting of data collection | Where was the data collected? e*.g. home, clinic, workplace* | Either at the hospitals (in-person) or via phone. |
| 15. | Presence of non-participants | Was anyone else present besides the participants and researchers? | No |
| 16. | Description of sample | What are the important characteristics of the sample? *e.g. demographic data, date* | See Table 1. |
| Data collection |  |  |  |
| 17. | Interview guide | Were questions, prompts, guides provided by the authors? Was it pilot tested? | Yes, the interview guideline was pilot tested at all sites, and the interview questions were provided to the participants in advance. |
| 18. | Repeat interviews | Were repeat interviews carried out? If yes, how many? | No. |
| 19. | Audio/visual recording | Did the research use audio or visual recording to collect the data? | The interviews were audio recorded. |
| 20. | Field notes | Were field notes made during and/or after the interview or focus group? | Yes, during the interview and reported within the interview form. |
| 21. | Duration | What was the duration of the interviews or focus group? | On average 35 minutes. See chapter 2.3. |
| 22. | Data saturation | Was data saturation discussed? | Yes, several times during the regular meetings among all study sites. |
| 23. | Transcripts returned | Were transcripts returned to participants for comment and/or correction? | No, but the results were discussed with stakeholders (patient representative, oncologists). See chapter 2.4. |
| **Domain 3: analysis and findings**z |  |  |  |
| Data analysis |  |  |  |
| 24. | Number of data coders | How many data coders coded the data? | 7; see chapter “author” contributions. |
| 25. | Description of the coding tree | Did authors provide a description of the coding tree? | Yes, see table 2 and 3 of the online resources and chapter 3.2. |
| 26. | Derivation of themes | Were themes identified in advance or derived from the data? | See chapter 2.4 |
| 27. | Software | What software, if applicable, was used to manage the data? | Qualitative data: MAXQDA Software (Release 18.2.4)  Quantitative data: Stata, version. 17.0 |
| 28. | Participant checking | Did participants provide feedback on the findings? | The results from the interviews were discussed with stakeholders (one patient representative, two oncologists). See chapter 2.4. |
| Reporting |  |  |  |
| 29. | Quotations presented | Were participant quotations presented to illustrate the themes / findings? Was each quotation identified? e*.g. participant number* | Yes, see Figure 1 and table 2 and 3 of the online resources. |
| 30. | Data and findings consistent | Was there consistency between the data presented and the findings? | Yes, see chapters 3.2, 3.3, and 4. |
| 31. | Clarity of major themes | Were major themes clearly presented in the findings? | Yes, see chapter 3.2. |
| 32. | Clarity of minor themes | Is there a description of diverse cases or discussion of minor themes? | Yes, see chapter 3.2. |

Allison Tong, Peter Sainsbury, Jonathan Craig, Consolidated criteria for reporting qualitative research (COREQ): a 32-item checklist for interviews and focus groups, International Journal for Quality in Health Care, Volume 19, Issue 6, December 2007, Pages 349–357, <https://doi.org/10.1093/intqhc/mzm042>
