# Supplementary material for: Patient-reported experiences of cancer care related to the COVID-19 pandemic in Switzerland
Source: Support Care Cancer. 2023 Jun 22;31(7):410. doi: 10.1007/s00520-023-07871-8 (PMC10287824; doi:10.1007/s00520-023-07871-8)
Supplement: Supplementary file 2 — (PDF 413 kb) [file 520_2023_7871_MOESM2_ESM.pdf]

Supportive Care in Cancer (JSCC)

Online Resource

Patient-reported experiences of cancer care related to the COVID-19 pandemic in Switzerland

Sara Colomer-Lahiguera*#, Claudia Canella#, Stellio Giacomini, Kim Lê Van, Carla Pedrazzani, Matthias Naegele, Laure Thouvenin, Alix O'Meara Stern, Rosaria Condorelli, Tourane Corbière, Claudia Witt, Manuela Eicher, Karin Ribi

# These authors contributed equally/shared first authorship

Corresponding author:

Sara Colomer-Lahiguera
Institute of Higher Education and Research in Healthcare (IUFRS)
Office 01/169 - PROLINE - Rte de la Corniche 10 – 1010 Lausanne
sara.colomer-lahiguera@chuv.ch

Supplementary Table 1 : interview guide

The interview form included the following open-ended questions:

1. Did the COVID-19 pandemic have any consequences related to your cancer? If so, what were they, and how did they manifest?
    a. What were the changes or delays related to your cancer treatment or your follow-up appointments for cancer care?
    b. Were there any changes in your experiences (e.g. lock-down vs no lock-down, getting used to the situation) over the different phases during the pandemic
    c. Which aspects of your health care were affected by COVID-19?
    d. How did you experience the social support (e.g. family, friends and larger social network)?
2. Do you feel at particular risk to get infected?
    a. If yes: Why do you feel at particular risk
    b. If no: Why do you not feel at particular risk?
    c. If you should get infected, do you feel at risk to develop severe events?
3. What do you think about the vaccination in your specific situation?
    a. What are your expectations regarding the vaccination in your specific situation?
4. What are the worries or needs you have related to the COVID-19 pandemic?
    a. What are the worries related to your cancer disease?
    b. Who or what helps you most to face or manage these worries?
    c. Is there anything that you would expect from your cancer care team to help you to manage these worries?
5. What are the needs you have related to the COVID-19 pandemic?
    a. What are the needs related to your cancer disease?
    b. Who or what helps you most to face or manage these needs?
    c. Is there anything that you would expect from your cancer care team to help you to manage these needs?
6. Is there anything that you perceive as positive change in the current situation during COVID-19 pandemic?

Supportive Care in Cancer (JSCC)

ESM_Table 2: Themes, subthemes, codes and quotes[1] from the three different language regions

THEME	Theme definition	Subtheme	Subtheme definition	Codes	CH_FR	CH_DE	CH_IT
PSYCHOLOGICAL DIMENSION	feelings and perceived risks regarding the COVID-19 pandemic, both personal and related to interaction with others	COVID and Cancer	Any experience of a psychological or emotional state related to COVID and cancer (positive or negative)	COVID and cancer	*It's true that sometimes it's more difficult when you're ill. I felt like that. But without that, psychologically, I didn't have too many problems, I must say that. It went really well.*	*Yes, well, it's like that, of course, like again, you had additional fear. Or? So, now one has actually already a disease, where one lives also always a little bit in fear...or not? And the Corona time has been of course also a threat again. (...) So like a double threat, so to speak.*	*So, it's a bad disease in itself. This is the impact, you live it very much alone, because you go alone to the therapies, to the consultations, apart from the first one where fortunately my husband accompanied me because there was permission, but you are always very alone. In addition, you live in isolation (...). So it's a struggle in loneliness and that weighs.*
		Risk of infection	Any statement about thoughts, fears, feelings, behaviors, etc. regarding therisk of getting infected or infecting others. Also included are statements about benefit-risk trade-off of a possible infection	Risk of infection	*I am more fragile than others.*	*Yes, so during the chemo period, also in the first two or three months, an acquaintance of friends of ours was abroad and she made her apartment available so that I could have asylum there, chemo asylum. So I went there for days at a time, when it was possible, just to have a bit of distance and a bit of peace. And on the way there, there was a store and I felt like having an ice cream. And I really had a guilty conscience that if I went in there now, I would catch Corona. And then I thought, is an ice cream really worth getting Corona now...So every time I've approached people I've really thought I'm risking my life. Or is it worth it to risk my life now.*	*And the fear of catching the virus: I used to say 'I'm weak, and if I catch this thing on top of that, it's over,' so that fear, yes, I had it. (...) The fear that because I was weak, I wouldn't make it. That was the fear I had.*

SOCIAL DIMENSION	personal, social, and political circumstances that result in experiences affecting daily life and well-being during the pandemic	Preventive measures	Any measure taken to prevent COVID infection.	Hygienic measures	*Yes, because everybody was paying attention. I always had to have the mask on and everything. My kids also, when they came, they put the mask on. It was strange at first but you get used to it.*	*At the beginning, I noticed that people didn't take the pandemic so seriously, even in the doctors' offices. Then I noticed that in part, even with the mask, that was also the case, that I had the option to remove the mask, for example. And then, I think, it became compulsory to have the mask on, and I noticed that people became more careful, also on the patient side, so that people took more distance, that more people wore an FFP 2 mask, that people disinfected their hands more regularly. At the beginning it wasn't like that, I didn't notice it that way, but then, yes, during the therapy I noticed that the measures were implemented much more strictly, in the practice, in my oncology practice or in the hospitals.*	*I live on disinfectants and masks. Even before, because I've always been very careful. When the thing came along even more so.*
				Social distancing	*Well during that time, what I can tell you is, we have five grandchildren and not being able to share meetings with them, it was very hard, very hard, yeah. That's the feeling I had with that experience.*	*So, we've been in it for a year now. And in the beginning I was very afraid, I isolated myself additionally. And at some point I was like saying: Okay, this is like something worse now. Do you not want to see your friends now, maybe in your last months, because of the COVID? Or do you still not care about that either? And at some point I had to say, okay, I don't care too much.*	*In the sense that no longer having any defense of my own, or at least quite low, I was quite afraid that I could catch it easier, so precisely zero social life.*
				Lockdown	*I went through the lockdown like everyone else, well with varying degrees of positive appreciation, but nothing really changed. I went through the lockdown and then the COVID like everyone else, let's say.*	*I got my cancer diagnosed two weeks before the lockdown. But I would now say that the Corona crisis, the lockdown, was actually more of a blessing than a curse for us.* [Because of the chemotherapy] *I had to completely isolate myself anyway because of the immunosuppressing effect. And with me, also my whole family. And due to the fact that my husband was then in home office and the childcare was closed and so on, quite a lot of risk factors for me actually fell away and I could actually start quite well. You actually hardly had to clear any stones out of the way, I'll say. Yes, it all worked out. This may sound stupid now, but it all fell into place quite happily.*	*I think we were a month and a half at home, more or less, and we couldn't even go out for groceries. That weighed on me because, even going to the (shop), nearby, was to move a little bit, but you couldn't.*
				Physical contact	*It went so far as to ask the question if we were going to celebrate the end of the year together or not. So at that point, we were really taking precautions regarding the distancing, it*	*What I also see (...) is simply that people no longer touch each other. And I just work with small children. And so that's very strange.*	*I am also careful when I am out and about to keep my distance, not to touch my eyes, mouth, nose with my dirty hands, without being phobic. I know that, all the more so in my case, I have to be even more careful.*

					is clear that we don't kiss each other anymore, in short, we are very careful.		*Clearly, if I were to take it (the Covid), the worry would be complications.*
		Regular activities	All kind of daily life activities that were affected by COVID-19	Sports, exercice	*Listen, the only thing is that I really liked going to the pool. Well, that wasn't possible, because it was closed. I missed that a lot. And the only thing I do is walk quite a bit. So when I could walk, I could go by myself, walk a little bit, whether it was for five minutes or not, but I did it. But always by myself.*	*Another important point about sports and exercise. The gyms were closed, of course, and it's very important to exercise and do a lot of sports during therapy. And I also found that difficult, because I'm more of a, I like to go to the gym, or I liked to go to the gym (laughing), also like to go to group classes and that was just, yeah, my workout. And, yes, during the pandemic that's just not possible and especially when the immune system is just not so good anymore (...) And then I had to switch to other sports, such as, yes, I went hiking a lot or started jogging, but that was also a change.*	*I missed the movement, because when I do chemo it is very important for me. Yes, I walked back and forth three or four times a day, twenty minutes or a quarter of an hour, but it was not enough.*
				Shopping	*Obviously we could not go shopping, we could not go to certain restaurants for a while but otherwise, life followed its course.*	*I'm not afraid, but I'm very, very careful, so I don't catch it. So, I still wear FFP2 mask and only meet with people, with selected people to go for a walk, and I order online.*	*Over the summer, when they then reopened last year, I went maybe two or three times to get other things, but I didn't buy clothes anymore, I didn't buy shoes… I was very limited to the essentials that I needed.*
				Mobility	*The only thing that stopped me from going out, from living a little bit as usual, it was the mask, and not taking the buses or trams and things like that. Yes, [COVID] prevented me from living a little bit too, from not going to the restaurant.*	*I tried to get there by bike whenever possible, because I didn't want to use public transport. But then that didn't work in some cases, where I was too ill with chemo.*	*When I am on the bus, I keep my distance or even try not to take the bus at all, I try to walk more.*
				Other activities	*There were a lot of things we couldn't do anymore, but it didn't particularly impact me, at least not for my cancer.*	*I can sing more than before, I have much more time for that. You've had less traffic noise, less airplane noise. Just things like that, which I appreciated very much (…) everything is a little bit lowered. I also find it quite lively when things don't run according to a timetable.*	*Travelling is my escape. Clearly, I was very limited during my illness, partly because everything was closed and you couldn't do much, partly because I was undergoing treatment. Last year I was only away at the end of treatment, a few days, otherwise I did nothing. (…) As long as I was in treatment, I didn't even feel like going too far; now that I'm finished, which in theory I could be a little safer, the situation is still unstable, so I don't want to go too far.*
				Child care, family life	*I don't have any grandchildren, but I have many nieces and nephews. In three families, I took care of the children, I took turns taking them, so I'll be happy when I can take them to sleep again or take them for a day or two like this.*	*Actually everything worked out quite well, it fit together. And because my husband went into short-time work at some point, he had two days off a week, and I was able to use these two days for myself and my therapy, so the children were taken care of (…) we were able to manage everything out of ourselves. Of course, that was also quite hard in the meantime.*	*My husband always went to the office, in the sense that he had his own company, so he had to be there and this perhaps saved the whole thing. I and my son, he in distance learning, I working from home, we found a good balance. So this brought us together and I was calm, because the child was at home. I had a bit of control. That fortified us. I know that many families have*

						But because somehow everyone/ we were all in the same boat, somehow it was clear.	*fallen apart, but not us. We did well, fortunately.*
				Work	*I'm lucky enough to work, so that leaves me no time to think (about cancer or COVID) too much , I go on with life and then come what may, as they say*	*What was also positive, I worked during the treatment, and I worked in home office, that is, I did not have to go to the office. That made a lot of things easier for me, because I could then also lie down when I was tired, or simply also when the hair then falls out, it is simply not so noticeable that you are sick and you can still participate in everyday work. And that was definitely something positive and it still is, because I have now also started to work again.*	*Working so much with people, even if there hadn't been the pandemic, I would have taken sick anyway, all the more so because, working closely with clients, it was not possible to continue.*
		Social interactions	Any statements regarding the impact of COVID-19 on social interactions	In-person interactions	*I'm so used to always being in contact with people, going out and everything, and then I found myself all alone, all by myself. It was hard.*	*You don't have to justify yourself if, for example, you can't be at activities now or something. I think that was also something positive, you have the feeling that life stands still anyway and you don't do much with friends anymore anyway, you can't go to the movies anymore or go on dates or something, and everyone actually feels the same way. And then the focus is not so much on the cancer treatment that you are currently undergoing, that is, for other people. It's not so noticeable and then it's also a little bit easier.*	*Besides the illness, you live in isolation, because apart from my husband, my son and a cousin who helps me, I don't see anyone. I haven't seen my best friend for seven months. So it is a struggle in loneliness and that weighs.*
				Digital communication	*I focused more on others while virtually keeping friends on the phone or FaceTime*	*I have to say that's great, because now I'm just thinking about it or discuss it* [a question] *with the doctor (…) by phone instead of going there, and she also does that, so I think that's great. I think that was not an option before and now it really is an option, so for me it's a super time saver...And I think also for the doctor. She calls me, then we talk and so I think it's certainly more effective in that sense. Of course it's good when the patient comes, but I think when you briefly compare blood levels or she lets me know blood levels are good, I think for her it's then also, yes efficiently done so with a phone call.*	*Technology has helped me. I am not a user of social media, in fact I detest it. Let's say the WhatsApp world has helped me. I have these support groups of my best friends. We talk through technology on the phone. Here there is no physical contact, sometimes you really need a hug.... Technology helps you feel a little less alone though... I miss it, just the human contact.*

		Back to normal	Any statement referring to life before COVID-19	Back to normal	*Now I wish, like everyone else, that we could go back to a really normal life soon. Right now, I feel I'm in the same situation as the majority of people.*	*I simply realize now, because I didn't do that last year, when there were the ease of the measures. Then I thought that it would stay like that and everyone went on vacation and I just realized, well, no, I absolutely have to tackle that now, just for me. So now I have to see if it's possible with therapy or something… but that would be such an important need for me to relax and have a positive experience, going to the sea and yes, a bit of normality.*	*I'm worried about that, because you can't give the final push, as they say on TV sports programs, to this disease. I'm worried about that because, little by little, with all the variants, etc., there is a risk that something worse or more difficult to fight may turn up, even though studies are increasing and are more precise on these things. That's a bit of a worry, of no longer having a normal life without this virus.*
		Society	Any statements related to COVID that concern changes or observation on a society level	Solidarity	*At the beginning everyone applauded the doctors and the nursing staff and then at the end they were told: "You just have to do your job, you're paid for it". So there's not much respect for the people in the hospitals.*	*People have been so busy with this pandemic and then to get a message or the message from someone: 'I have cancer now and I'm doing chemo'. They've been so blindsided and if there's an emergency situation like that, an extraordinary situation like that, then that's bad news. (…) And I think it affected many, even more than it would have otherwise, because they were somehow in a state of alarm. And many people who really wanted to do something for us, wanted to help, big solidarity. Especially with Corona, which was of course difficult.*	*What also helped me a lot is that my mom, for example, told me 'See? Everything is closed now, so you have to be careful, you have to stay at home, but the others have to stay at home too, so they are all in solidarity with you'. Definitely that helped me because when you can't do anything and you see others doing everything, it's a little frustrating. So instead, for me, it was kind of like 'We're all in the same boat'.*
				Peoples' behaviour	*[…] before, when we saw people without masks, it felt like an aggression, during shopping or outside.*	*I already experienced scenes in public transport, where a person was really roughly approached, because they don't have a mask, although you don't even know whether they are somehow really exempted by the doctor. But really roughly! And that is coupled with fear. The people who are afraid and therefore behave a bit strangely, be aggressive or whatever.*	*Every so often when I saw these big gatherings, it bothered me but I couldn't do anything about it.*
SUPPORT DIMENSION	resources used to deal with the pandemic	External resources	Any statements that relate to a support received by an informal caregiver (family, friends, acquaintances or neighbors), community or non-institutional support	Friends support	*I had a WhatsApp group that I had created with my closest friends and my family, and they were always available to encourage me. So I'm very well supervised and I think it also helps my morale.*	*I still got a lot of support from friends and family and, for example, I was always picked up from chemotherapy by a friend. So, every week she waited for me outside the practice and brought me home.*	*I have a lot of friends. I was on the phone a lot, because if it was one or the other, and then if I needed to talk, I would call too. In that sense, I never felt alone. Even when I'm well.*
				Family support	*But I am well surrounded, my wife is behind me to boost me a little bit from time to time. So there are just the two of us. If I was alone, maybe it would be more difficult.*	*Of course, then also great support from my partner, who just always wrote to me via WhatsApp or called me via video or just looked to see if everything was okay. Also on the family side, I still have five siblings and they also agreed on who calls me. They always made sure that someone contacts me, just to show that they are*	*[My family members were] clearly, a little less close physically, but they always wrote to me, they always called me, they always asked. My mom came to bring me groceries, from the distance. Even though I was alone at home, I didn't feel lonely on a social level, they were really close to me.*

						there, even if they can not be with me now. And, yeah, to experience that support was very nice, or is very nice, so, I still get the support today.	
				Other resources	*And then what was also important and helpful was the library. There's a library in [City C] that organized things very well, at one point you could get things at home and then come and collect them by appointment.*	*I have received a lot of understanding from my employer, from my supervisor, until now and in my therapy as well. My employer has my back quite a bit, which I think is very nice.*	*I joined a group of ex-patients, so we already met a couple of times with them, and then also with a nurse, (...) I went for walks, which they also organized with other patients*
		Internal resources	Relates to anything the patients expressed helped them during the pandemic (state of mind, resilience, coping strategies, activities)	Instrumental	*About the fact that I went out less, I compensated for it with a lot of interest in my activities that I had set for myself.*	You had to be very creative; you had to come up with something when it wasn't just there. I came up with the most exciting ideas! (...) during the lockdown, somehow making sprout jars out of yogurt things with drills.	*I used to turn off the television because I didn't want to hear that stuff all the time. I wanted to relax, so I read, but I wasn't obsessed with reading everything I could on Covid. I would listen, rather, and afterwards I would relax by reading books or doing the crossword puzzle.*
				Emotional	*Maybe it's also the fact that the disease allowed me to deal with things one day at a time.*	*Sometimes it did me good to just wrap that up, close the "drawer", distract myself, make a joke again and just have normal conversations. Not like all about cancer and COVID. Right?*	*It was easier for us cancer patients to accept the lockdown. Because we already live in a similar situation. That is, living for the day because you are in a situation where you no longer make long-distance plans. I live to be well today and to be well tomorrow. If I'm well tomorrow, then I think maybe I'll do a week away from home. This is just to tell you, even the question, the reality death is something to consider. Whereas before I did not take death into consideration.*
HEALTHCARE DIMENSION	any situation related to changes in cancer care because of the pandemic, covering systemic and individual perspective	Cancer care delivery	Statements of a specific experience related to care during one's own cancer treatment under pandemic conditions. The experiences may have different characteristics and qualities, e.g., helpful, challenging, positive or negative.	Cancer centers reorganisation	*What was different was at the entrance to the hospital. They wanted to know about the appointments, if it was scheduled, or that you couldn't stay more than an hour or something like that.*	*I got the diagnosis with the lockdown. I witnessed how the hospital then became a military zone (...) there were checkpoints everywhere. The procedures were different every week. First the temperature was taken in one place and then in three or four other places. Then there was a questionnaire. The next week it was a different questionnaire. Then you got a piece of paper that you had to take with you... And in such a situation where you don't know what the treatment will do to you, it's like an additional stress point. That already affected me. Or also when my husband couldn't come with me for the diagnosis meeting. To do that alone, that was already difficult, yes...We connected him by telephone. But it's just not the same if someone can't hold your hand.*	*I don't know how they normally do it, but clearly the change, for me, was that I had to go to [city B] to have chemo, instead of having it in [city A]. From March to June I had to arrange for someone to drive me.*

				Cancer center measures taken	*The only thing that happened was that I had to go alone, when my husband didn't accompany me, when my husband couldn't be with me, I think that's the only thing that made me sad.*	*The pandemic had an impact because of course I always had to go to the examinations alone. I was not allowed to take a companion with me, which was relatively difficult, especially at the beginning, when the diagnosis was made. Also during the discussion of the treatments I always had my companion with me via video conference and that is of course something different.*	*At the level of care, I don't know how it is normally, but I felt really taken care of. Clearly with all the precautions, they were very kind, they even gave me advice: they told me not to go out. I asked them all the questions that were needed, and they were very helpful.*
				Clinical team	*In relation to the care team, I would say that COVID did not have much influence, except for the sanitary measures and precautions to take. But the care team, my goodness, they saved me with a mask. They would not have received me with a mask if it had not been for COVID. But in terms of care, I had no problem. On the other hand, it's true that I always mentioned cancer in terms of increased risk in case of COVID, but that didn't change anything in terms of hospital care.*	*I thought that was not bad, especially in the first phase, when the operations were scaled down. The treating physician simply had more time and explained things better. She also accompanied me at times when she would otherwise have had no time. And that did me an enormous amount of good. Or just during the first chemo, the nursing staff also had more time to simply support you. It has not only been negative.*	*My oncologist gave me so many suggestions, she even got me articles at the beginning of the pandemic about the coronavirus, what to do, instructions, so I found a lot of support. Also in talking I found a lot of support.*
		Individual cancer trajectory / development	Statements that describe the consequences of changes in patients' individual disease/illness trajectory.	Individual cancer trajectory/development	*I was diagnosed and then came COVID, the first wave. I was hospitalized, so there was not a real interaction between the two [Cancer and COVID], but they came at about the same time.*	*In 2020 I had the control, after the beginning of COVID, there were quite a lot of metastases in the lungs again. I was suddenly locked up at home, I couldn't get out and I couldn't meet anybody except with mask and so. I had the feeling that this limited my soul, my motivation quite firmly. And then everything just started. Then again during the pandemic I had to start chemo. No one was allowed in, neither parents nor friends, which meant that I always had to go alone. Which then was additionally a burden to the whole thing.*	*For the treatment itself, nothing changed. The discomfort was just going to [city B]. I'm better off in [city A], and that's for sure, but for how they worked, nothing changed. The discomfort was going all the way there.*
VACCINATION	Perception and handling of public health recommendations regarding vaccination	Emotions related to vaccination	Statements related to vaccination, i.e. beliefs, expectations, hopes, fears, risk considerations, etc., both related to oneself and to other persons	Emotions related to vaccination	*Reassured first of all, to get it* [the vaccine]*, to be able to do it and then reassured because I received my vaccine on the 4th of February, I think, and I was very happy to feel more protected compared to the outside.*	*The fact that I was then vaccinated, yes, I have now like a security. I still protect myself the same way but I feel more liberated after the vaccination. I've also been told that you don't know how the drug works when you have chemotherapy now. But anyway, I just felt better, I felt more secure I must say.*	*I think it's helpful because that's the only way we beat the other viruses we've had.*

		Alignment with authorities	Includes statements/expressions about resignation, trust in politics, resilience, acceptance of the unavoidable, following advice from health professionals.	Alignment with authorities	*At the very beginning, I didn't want to be vaccinated. And then my oncologist told me: "No, no, but do it anyway for you, it will be much better. And then you'll see..." And well, I let myself be convinced. And then I did it. But frankly, I didn't want to do it at first.*	*I think it's very important. I have been double vaccinated for almost two months. I was skeptical at first because I was afraid that it was developed too quickly. That there hasn't been enough time to test it. But I think the media and the responsible federal offices have provided good information to convince people. That research on coronaviruses has been going on for a long time and that it was not research that only started then. But that it was built up long before and then it was no longer a question for me. My husband has also been vaccinated.(...) This has brought a great relief for me mentally. Somehow, now I think I have like such a protective shield. Although how long this protective shield will be of any use now is something else. But for me it hasn't really been a question.*	*Yes, but they all tell me to do it, clearly. I also expressed my doubts, and they said 'Look, it's more the benefits than the risks'. I don't know, I'm the one who, inside of me, when I had to do it I was like 'Wait, it's not time yet,' maybe because I had already been through all these treatments and I didn't want to add that, that maybe I would be sick from that too.*
		Consequences of vaccination	Statements about the beliefs of consequences of vaccination including the impact on individual, public health and non-health domains, symptoms, protection of self and others.	Impact on health	*[...] I believe that the vaccine already allows us to be protected ourselves and to protect the people around us, since if we are vaccinated, the possibilities of catching the disease are infinitely smaller and consequently the possibilities of contagion are also smaller.*	*And then there was another sign with the cancer that I had the expectation or the hope that I would not get Corona in this time when I am weakened. Or yes I would get Corona maybe a little weaker then, not in this pronounced form. Not two things that my body would have to master at once. As protection. That's then more as protection for me, really about me personally, that's why I wanted it quickly.*	*If I were to get sick, for some reason, I think it protects me and doesn't make me so sick, it protects me and doesn't make me sick at all*
				Impact on non-health domains	*I'm less afraid than before. And I still try to hang out with people who have already been vaccinated so that I'm not really in danger either, because we have friends who have already been vaccinated, so we hang out with those rather than with the others. The others, it's maybe only by phone.*	*So I have to say quite specifically, I've only been really relaxed since I did the vaccination. And even there I have not been really relaxed yet. So I am double vaccinated now. So I can meet friends again or just outside in a restaurant and that's now for three weeks.*	*I tell you the truth: I don't worry anymore. In fact, we went to [place], but I didn't worry because I used to put a mask on when we went into the restaurant, or so, and with the vaccine I didn't worry at all, I was calm.*
		Logistics of vaccination	Entails statements about the logisitics of vaccination in general : organisation, procedures, apointments, etc.	Logistics of vaccination	*The place where we were vaccinated, I went to the [Place D], it was superbly organised... we are well received and all that.*	*I have completed acute therapy and am now on anti-hormone therapy, and therefore I no longer belong to the risk group, which means that I now have to register online as normal and am now waiting for my vaccination appointment.*	*The lack of vaccines didn't allow us a serene, as they say, closeness. That was the thing. It was obvious one thing: that there was a lack of vaccines. So we put everything on standby, and for me it was resolved yesterday, because I was vaccinated.*

[1]The interviews were conducted in the native languages of the three regions: French, Swiss German, and Italian. The quotes were then translated to English by the authors.

Supportive Care in Cancer (JSCC)

ESM_Table 3: Transversal themes and illustrative quotes[1]

NEEDS	PSYCHOLOGICAL DIMENSION	*They also refer you to X places, where you can turn to, if you now have psychological problems or so. But maybe that's not always the case. Maybe it would be enough just to listen for five minutes.*
	SOCIAL DIMENSION	*For me it's more about (...) I don't know, meeting a good friend again and then saying hello. Not just throwing a little heart or a kiss, but just hugging each other again. And just that human contact…I would have liked to shake my doctor's hand in the breast center and just really like to say: "Thank you"…I don't know, this interpersonal thing, but also this physical thing…Or just sit in the café…and watch the people on the street. You can still do that, but I still don't dare…So these are such little things somehow. And (...) I would say that's what I'm missing now.*
	SUPPORT DIMENSION	*I have to say that it's true that I was weakened and that to this extent, my sister could help me from time to time, it was little things, it was small things. But on a day-to-day basis, I managed on my own and that was it. I didn't use any particular services or anything.*
	HEALTHCARE DIMENSION	*I think // simply a consistent implementation of the measures. That you do what you know you can do. Just distance, mask, hand hygiene.*
	VACCINATION	*Needs [inaudible] no. Yes, the only thing I could say is to be..., to keep being informed about the vaccine we were given, to have a follow-up to know how it went, how people in general feel, if there were any, how to say it, tests that proved that people who had cancer, whether they were much more affected or less affected or much more in a dangerous situation, not dangerous, well this kind of information would interest me to know in relation to covid.*

POSITIVE CHANGES	PSYCHOLOGICAL DIMENSION	*So positive change... I mean I felt like I was lucky in the bad luck that my cancer and my treatment fell in this pandemic period. Because if it's..., if I do this treatment before and everything, for example, I think it could be more of a source of frustration. Because of the fact that you're locked up, that you have to take care of yourself, that you can't travel, that you have to go to the hospital a lot, and with the fact that everyone was locked up, everyone had to take care, I didn't feel like I was in a very special situation with the treatment. It's a bit, it's a bit paradoxical but... there you go, it was somehow a positive effect. And good positive change is that I think everyone slowed down a little bit, so with the pandemic, me, I was very much around the house because my husband works from home.*
	SOCIAL DIMENSION	*And that you don't have to justify yourself if, for example, you can't be at activities now or something. I think that was, I think, also something positive, you have the feeling that life stands still anyway and you don't do much with friends anyway, you can't go to the movies or go on dates or something, and everybody feels the same way. And then the focus is not so much on the cancer treatment that you are currently undergoing, that is, for other people. It's not so noticeable and then it's also a little bit easier.*
	SUPPORT DIMENSION	*So I got my cancer diagnosed two weeks before the lockdown. So from that point of view, I didn't get cancer because of it. But I would now say that the Corona crisis, the lockdown was actually more of a blessing than a curse for us, because simply going hand in hand, simply through the therapy, which started quite quickly for me, a chemotherapy, I had to completely isolate myself anyway because of the immunosuppressing effect. And with me simply also my whole family. And due to the fact that my husband was then in the home office, the kindergarten closed and so on and immediately, quite a lot of risk factors for me actually fell away and I could actually start quite well. You actually hardly had to clear any stones out of the way, I'll say. Yes, everything/ this may sound stupid now, but everything fell into place quite happily.*

	HEALTHCARE DIMENSION	*Let's just say that the hospital, for me, was almost a social outing because it was the only thing I did outside of the house. I had a good relationship with the hospital, the nurses, the doctors and all the hospital staff, I felt a bit like visiting friends, in a certain sense, that is, they really helped me a lot. Clearly, in a period like this, when you couldn't see anyone, they were my only social contact outside the family, because even with my friends we didn't see each other, more than anything else because they too were afraid to see me, so as not to put me at risk, because anyway maybe they work with people, so it's a little more delicate.*
	VACCINATION	*Well, in Covid, the only positive thing that has happened to me is that I was able to get the vaccine. I feel like that's maybe a little bit of an extra precaution that makes me a little bit safer from this Covid.*

PHASES OF THE PANDEMIC	PSYCHOLOGICAL DIMENSION	*At first, I was so afraid because I had just started chemo last year, in March. I was afraid because I was weak and went out very little. Then, people over 65 couldn't go out, and for a month and a half I just didn't move from home. I struggled because I needed to walk. It was this disorder here, and the fear of going out, because I didn't want to take it. Apart from that, it went away. When I was able to go out, I already felt calmer, because I went for a walk every day and I really felt much better.*
	SOCIAL DIMENSION	*So in the beginning I was just happy that everything was so/ so, that it just fell into place, as already mentioned. And then, however/ in the summer, when it became a bit looser and in the fall also, through that/ Well, I have an older son who goes to kindergarten and he then had to continue to be looked after in homeschooling, even after the kindergarten had opened again. That was a bit hard, because you just noticed that outside, life is awakening again. People are walking more again, there's just more going on in the streets and so on. And we still had to isolate ourselves, which was hard at times to make him understand.*
	HEALTHCARE DIMENSION	*During the first wave, I was being followed up on the phone, making video calls with my oncologist and no longer going to appointments. This meant that I didn't even do blood tests, and unfortunately, right around this time, I had a bit of a cough, but this fact of not going to check-ups, of not being watched live, did affect me a bit in the sense that I then ended up in hospital.*
	VACCINATION	*And then it slowly came up with this vaccination. And then, now, there are three phases. Now, I'll say about three or four weeks ago, most of the people around me have actually already been vaccinated. And also many of my friends and my children, so my son and my parents. And from that, yes, it has now already loosened up a bit, so that it somehow like a little bit open again. But I'm just not yet so well that I somehow could benefit from that.*

[1]The interviews were conducted in the native languages of the three regions: French, Swiss German, and Italian. The quotes were then translated to English by the authors.
